# Platelets’ RNA as biomarker trove for differentiation of early-stage hepatocellular carcinoma from underlying cirrhotic nodules

**DOI:** 10.1371/journal.pone.0256739

**Published:** 2021-09-01

**Authors:** Walifa Waqar, Sidra Asghar, Sobia Manzoor

**Affiliations:** Department of Healthcare Biotechnology, Atta-ur-Rahman School of Applied Biosciences, National University of Sciences and Technology, Islamabad, Pakistan; Texas A&M University, UNITED STATES

## Abstract

**Background & aims:**

Among the multiplicity of factors involved in rising incidence of hepatocellular carcinoma (HCC)-the second deadliest cancer, late diagnosis of early-stage HCC nodules originating from late-stage cirrhotic nodules is the most crucial. In recent years, Tumor-educated platelets (TEPs) have emerged as a strong multimodal tool to be used in liquid-biopsy of cancers because of changes in their mRNA content. This study assessed the reliability of selected mRNA repertoire of platelets as biomarkers to differentiate early HCC from late-stage cirrhotic nodules.

**Methods:**

Quantitative real time PCR (qRT-PCR) was used to evaluate expression levels of selected platelets-specific mRNA between HCC patients compared to cirrhosis patients. ROC curve analysis assessed the sensitivity and specificity of the biomarkers.

**Results:**

RhoA, CTNNB1 and SPINK1 showed a significant 3.3-, 3.2- and 3.18-folds upregulation, respectively, in HCC patients compared to cirrhosis patients while IFITM3 and SERPIND1 presented a 2.24-fold change. Strikingly, CD41+ platelets also demonstrated a marked difference of expression in HCC and cirrhosis groups.

**Conclusions:**

Our study reports liquid biopsy-based platelets mRNA signature for early diagnosis of HCC from underlying cirrhotic nodules. Moreover, differential expression of CD41+ platelets in two groups provides new insights into a probable link between CD41 expression on platelets with the progression of cirrhosis to HCC.

## Introduction

Hepatocellular carcinoma is the second major death-causing malignancy worldwide with a continually rising incidence [1]. Hepatic cirrhosis is a chronic liver disease in which normal liver cells are replaced with scar tissue. It progresses over the years or decades and the late stage where the liver has sustained considerable damage, it is referred to as decompensated cirrhosis or decompensated chronic liver disease (DCLD) [2].

Hepatocellular carcinoma (HCC) and cirrhosis have become a marked threat to global health in the recent years. Combined, these two pathologies account for 3.5% of all the deaths worldwide [3]. The major reason for HCC being one of the least curable malignancies is its poor rate of survival, mainly due to the diagnosis of this cancer at later stages when only palliative treatment can be given [4]. The high frequency of HCC is also due to poor prognosis and high mortality rates as well as limited access to treatment in underdeveloped areas.

While different etiological agents are responsible for the incidence of HCC, cirrhosis is one of the leading causes and HCC is among the major causes of deaths in patients with cirrhosis [5]. Therefore, the differentiation of HCC modules from underlying cirrhotic nodules with the perpetuated HCC is of prime importance in order to opt for the suitable curative treatment and liver transplant strategies.

Currently, some of the tools used in HCC diagnosis are the costly computed tomography (CT) scan, ultrasound (US), magnetic resonance imaging (MRI) as well as the blood biomarkers such as Alpha fetoprotein (AFP) [6]. According to a meta-analysis of surveillance imaging and AFP for earlier HCC diagnosis, ultrasound detects early-stage HCC with only 47% sensitivity (95% CI 33%-61%) while along with AFP, it detected early-stage HCC with 63% sensitivity (95% CI 48%-75%) [7]. AFP is not a good marker for diagnosing small HCCs at early stages and its levels are increased in case of HCV-related cirrhosis even in absence of HCC [8]. Liver biopsy is still the gold standard for diagnosis of various chronic liver diseases but in patients with cirrhosis, it is difficult to detect HCC at early stages with greater specificity and higher sensitivity with currently available markers [9].

There are no specific markers for differentiation between late-stage cirrhosis and early-stage of liver cancer (specifically HCC) and 40% false negative rate is reported in HCC detection [10]. This emphasizes the need to explore new reliable biomarkers for early diagnosis of HCC and its differentiation from underlying cirrhotic nodules to enable timely treatment of HCC before it becomes unmanageable. The increasing number of novel approaches for the establishment of liquid biopsy and the search for noninvasive biomarkers entails scrutiny of every possible entity that could fit the required characteristics of an ideal biomarker.

In the recent years, platelets studies have gained global attention for being used as a liquid biopsy tool to replace the invasive tissue biopsy and other nonspecific tools. Majority of the RNA transcripts are acquired from megakaryocytes during platelet production as platelets themselves are devoid of nucleus [11]. Besides their normal role in maintaining homeostasis and helping the body, it has been demonstrated that platelets interact with cancer cells either directly or indirectly via signaling molecules and affect tumor growth and dissemination. This interaction alters the mRNA as well as protein content of platelets as they uptake various microparticles during interaction with other cells or their surrounding environment [12].

Moreover, platelets ingest several biomolecules that are released by the tumor cells leading to education of platelets by cancer cells. These altered platelets are referred to as ‘Tumor-educated Platelets (TEPs)’ that promote the tumor progression and other hallmarks of cancers [13]. TEPs are predominantly affected by tumor types hence they can have an impactful role in cancer diagnosis [14]. In a study by Best et al., mRNA sequencing of TEPs has been reported to have 96% accuracy in differentiating patients with tumors from healthy individuals. TEPs also enabled pinpointing the location of six types of tumors (colorectal cancer, glioblastoma, hepatobiliary cancer, breast cancer and pancreatic cancer) with 71% accuracy [15].

The RNA repertoire of platelets that have interacted with cancer cells is modified and this property can be exploited for early diagnosis of HCC. Previously, our research group utilized tumor-educated platelets (TEPs) for early stage detection of HCC from healthy controls [16]. The rising incidence of HCC stems from its late diagnosis when it is developing on cirrhotic background, which accounts for 70–90% of all HCC cases [17]. Hence, in the current study, we used a similar approach but focused our efforts to analyze the role of platelets RNA as a prospective biomarker for diagnostic comparison of late-stage liver cirrhosis nodules and early-stage HCC. We assessed the differential expression of selected potential mRNA biomarkers among HCC and cirrhosis patients. The prospective biomarkers assessed in our study included CTNNB1, SERPIND1, SPINK1, RhoA and IFITM3. These mRNA biomarkers were selected based on their known role in HCC hallmarks, various aspects of progression of cirrhosis to HCC, initiation of cancer development, HCC proliferation and metastasis which could potentially be used in clinical management of liver cirrhosis and prevention of HCC. To the best of our knowledge, this is the first study analyzing the gene expression profiles of cirrhosis and HCC patients for their comparison while utilizing platelets’ mRNA profiles as a means of liquid biopsy.

## Methodology

### Ethical approval and sample collection

The approval for study protocol was taken from Internal Review Board (IRB) of the parent department Atta-ur-Rahman School of Applied Biosciences (ASAB), National University of Sciences and Technology, Islamabad, Pakistan. For blood samples collection, IRB approval was obtained from the Ethical Committee Board of Rawalpindi Medical University (RMU), Pakistan. Our study involved two experimental groups, i.e. cirrhosis and early-stage HCC patients. Samples for both groups were collected at Center of Liver Disease (CLD) and Gastrointestinal Ward of Holy Family Hospital (HFH) Rawalpindi, Pakistan. Informed written consent was obtained beforehand from the study participants. The study was carried out in accordance with the principles of Declaration of Helsinki.

All of the collected samples were processed at Atta-ur-Rahman School of Applied Biosciences (ASAB), National University of Sciences and Technology, Islamabad, Pakistan, within 2–3 hours of sample collection.

### Inclusion exclusion criteria

Patients with confirmed HCC having non-metastatic stage, and patients with confirmed cirrhosis, both verified by consultant gastroenterologists at aforementioned CLD-HFH, were included in the study. The disease status was confirmed via CT scan and histopathological analysis. HCC staging was done in accordance with Barcelona-Clinic Liver Cancer (BCLC) by the consultant gastroenterologist as routine procedure. Any unconfirmed case of HCC or late-stage (C or D) HCC as well as those having metastasized cancer were excluded. Categorically, 20 HCC patients and 20 cirrhosis patients with an age range of 32 to 85 participated in this study. 10 age-matched healthy controls with normal liver profile were included that do not have any history of liver disease or any form of cancer. They were also tested to be negative for HCV and HBV, confirmed via Immunohistochemistry (IHC) strip test, to avoid inclusion of any undetermined cases of hepatitis induced cirrhosis or HCC.

### Platelets isolation

Isolation of platelets was carried out from the collected blood samples using density gradient centrifugation for fractionation of blood. Pure platelets population was then obtained from the platelet-rich plasma (PRP). 6ml of the collected blood was subjected to centrifugation at 120g for 20 min. The acceleration and deceleration speeds of the centrifuge rotor were kept zero in order to avoid disturbing the pellet. This centrifugation step separated the red blood cells and a buffy coat layer from the desired platelet rich plasma (PRP). 2/3 of the uppermost PRP layer was collected, transferred to a new tube and centrifuged at 360g for 20min. Supernatant was carefully aspirated to avoid disturbing the platelet pellet. The obtained cell pellet was resuspended in 100μl of ACDA buffer before being subjected to RNA extraction.

### Flow cytometric analysis

For confirmation of platelets isolation, flow cytometric analysis of obtained cells was carried out using the platelet specific CD41 marker. CD41 marker (anti-CD41 antibody, MA1-12478 Thermoscientific) was first conjugated with FITC fluorochrome. Platelets were then incubated with anti-CD41 antibody for 1hr and subsequently washed with 0.1% PBS/BSA. Paraformaldehyde was used for fixation of platelets which were later analyzed using BD FACScan flow cytometer. Morphological gating was done based on the forward scatter and side scatter and mean fluorescence intensity (MFI) was evaluated. Data analysis was carried out using CellQuest BD FACScan.

### RNA extraction & cDNA synthesis

The RNA was extracted from isolated platelets of infected and non-infected participants using MagMax mirVana Total RNA isolation kit (Cat#A27828) according to manufacturer’s instructions. cDNA was synthesized from this extracted RNA using Thermo Scientific’s RevertAid First strand cDNA Synthesis Kit (Cat#K1622). The quality of cDNA was checked by doing PCR with housekeeping β-actin gene and running the products on 2% agarose gel.

### Real time quantitative PCR (qRT-PCR)

In order to analyze the expression of selected biomarkers in our study groups, primers for SERPIND1 and SPINK1 were designed using NCBI published sequences of human gene transcripts and primers with highest specificity were selected. *F*: *CATAACATCTGCGTGGGGTG* and *R*: *CCAGATAGTCGTCGTCCTCC* primers were used for amplification of SERPIND1 while SPINK1 was amplified using *F*: *GTCAATCAATAACCAGGGAGA* and R: *AAGGCACTGAGAAGAAAGATG*. The primers β-actin, IFITM3, CTNNB1 and RhoA were taken from published literature [18–21] and their primer sequences are given in S1 Table.

Quantitative real-time PCR (RT-qPCR) was performed using a 5X HOT FIREPol EvaGreen qPCR Mix Plus (ROX) in a total volume of 20μL with cDNA, primers and 40 cycles of PCR. The specificity of the amplified products was confirmed by visualization on a 2% agarose gel. β-actin gene was used for normalization. For quantification of gene expression, the comparative CT method (2^−ΔΔCT^) was utilized.

### Statistical analysis

All the results of flow cytometry are presented as mean ± SD while results from RT-qPCR are expressed as mean ± SEM from at least duplicate experiments. Statistical analysis was performed using GraphPad Prism version 5.0 (GraphPad Software, Inc., CA, USA). Data of gene expression levels has been analyzed using two-tailed t-test or two-way ANOVA where applicable, with 95% Confidence Interval (CI = 95%). The receiver operating characteristic (ROC) curve was plotted to determine the diagnostic accuracy of HCC biomarkers used in the study. Diagnostic parameters such as sensitivity, specificity and area under the curve (AUC) were also calculated with 95% Confidence Interval. *p*-values of <0.05 are considered to be statistically significant for difference of fold change in gene expression. Statistically significant data is indicated by asterisks (*p<0.05, **p<0.01, ***p<0.001).

## Results

### Patients’ characteristics

For this study, samples were collected from 10 healthy subjects and 40 patients among which 20 had liver cirrhosis (16 males, 4 females with age range 52.1±11.7 [mean ± SD]) while 20 had HCC (13 males, 7 females with age range 61.9±12.03) on the background of liver cirrhosis. The number of males in our study were more as compared to females, corelating to the predominance of cirrhosis and HCC in males, all around the globe having different ratios in different geographic regions ranging between 2:1 to 8:1 [22]. Among the HCC cases, 17 were HCV-mediated while only 3 were HBV-mediated, and 14 cases of cirrhosis were mediated by HCV infection, 2 were HBV-mediated and 4 had cryptogenic cirrhosis. This correlates to the fact that most common etiology of cirrhosis and HCC in Pakistan is HCV [23].

### Flow cytometric analysis and differential expression of CD41

**Fig 1:**
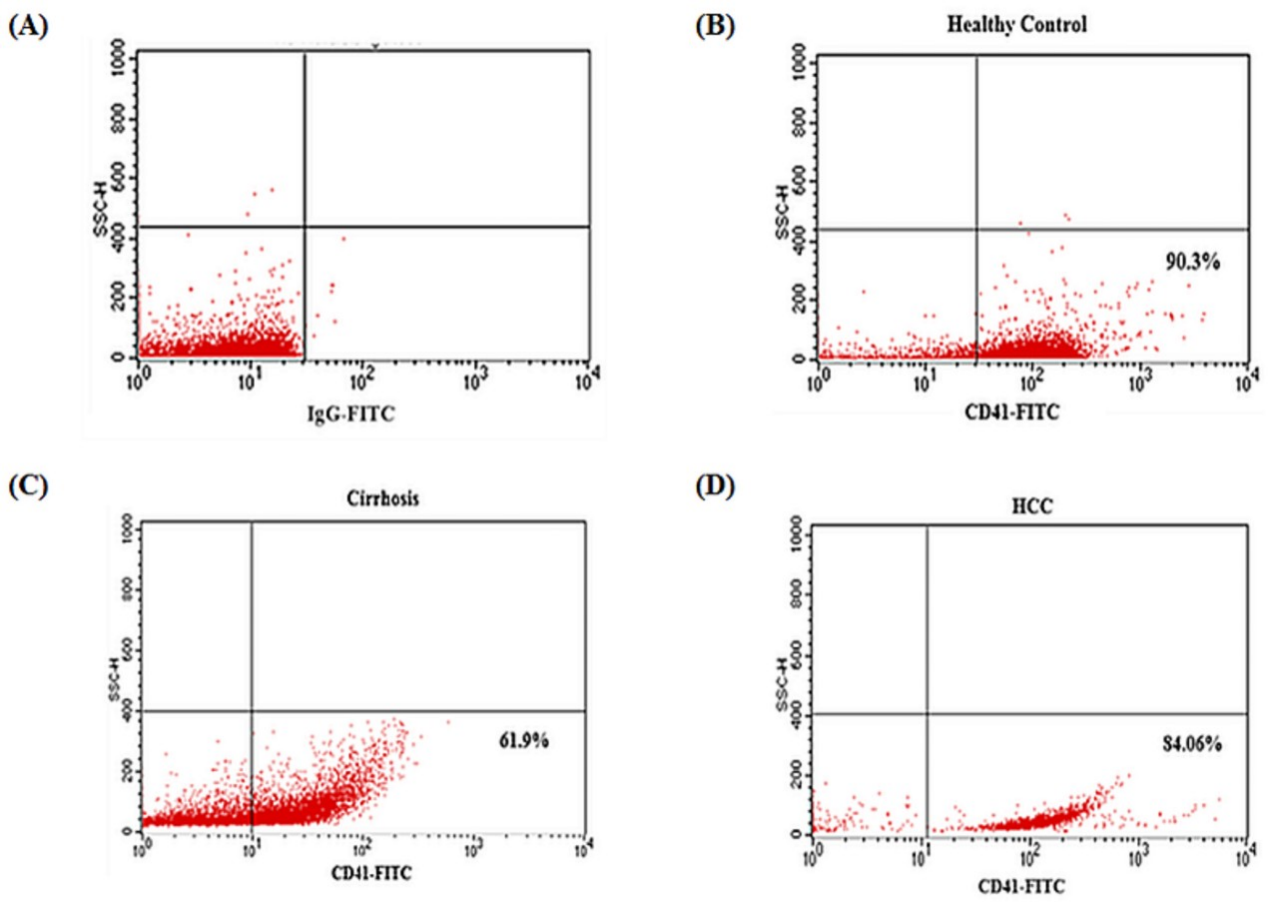
Flow cytometric analysis of CD41-stained platelets. (A) shows IgG-FITC used as a control while (B), (C), (D) show flow cytometric dot plots of expression of platelet membrane marker CD41 in the platelets derived after density gradient centrifugation. (B) CD41-stained platelets of healthy control. (C) CD41-stained platelets of cirrhosis patient. (D) CD41-stained platelets of HCC patient. CD41-FITC+ platelets are evaluated based on SSC/log FLH-1 where FLH-1 = CD41-FITC. The percentages given in lower right quadrant show the CD41^+^ cells in gated platelets population.

Platelets were identified based on their Forward Scatter (FSC) and Side scatter (SSC). Gated platelets were further analyzed for their CD41 expression and MFI as shown in Fig 1.

Around 80–90% population of CD41^+^ platelets was seen in gated events (platelets) from healthy controls as well as in HCC patients while striking difference with marked decrease in case of cirrhosis was observed where it was as low as 61.9–66%. The percentages of CD41^+^ platelets and their MFI are shown in Table 1.

**Table 1:** CD41+ platelets and their mean fluorescence intensity (MFI).

Sample	CD41^+^ platelets (%) ± SD	MFI ± SD
**HCC**	82.2 ± 2.5	184 ± 38
**Cirrhosis**	63.9 ± 2.8	31 ± 4.2

**Fig 2:**
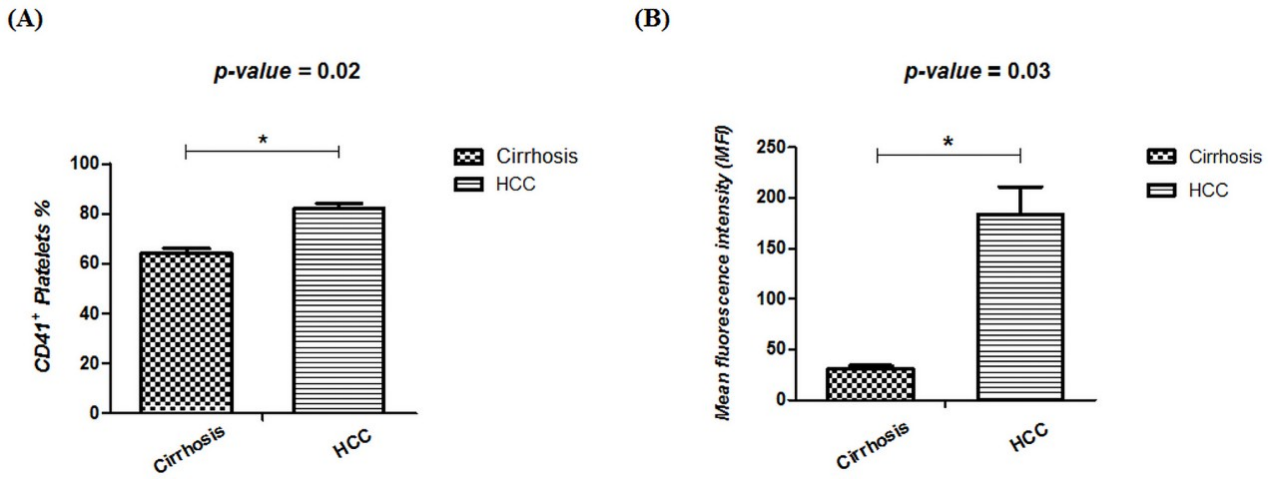
Graphical representation of percentages of (A) CD41+ population in platelets obtained from healthy control, cirrhosis and HCC patients. Statistical analysis using t-test yielded significant p values for difference in cirrhosis and HCC patients (*p<0.05). (B) MFI of CD41 expression in platelets of cirrhosis and HCC patients. Statistical analysis of MFI also gave significant *p value<0.05 for difference between cirrhosis and HCC patients.

Our results depicted that percentage of CD41^+^ platelets correlate with the disease status i.e. decreased percentage in cirrhosis while increase in percentage in case of HCC patients (Fig 2A). One particular case of a patient with suspected/very-early-stage HCC had around similar percentage of CD41^+^ platelets i.e. 81.4% that is in accordance with our results of early-stage HCC but, it showed reduced MFI i.e. 41, which is near to the MFI of CD41 expression in cirrhosis patients. This interesting finding can lead to further hypothesis that as the disease progresses from cirrhosis to HCC, there is an increase of CD41 expression on the surface of platelets.

This study also evaluated the MFI (Fig 2B) which demonstrated a remarkable decrease in CD41 expression on platelets isolated from cirrhosis patients depicting a striking difference from HCC. This allows us to suggest a probable association of CD41 expression and disease progression from cirrhosis to HCC.

### Gene expression analysis in cirrhosis and HCC patients

The expression of selected set of genes in HCC patients was compared with their expression in cirrhosis patients in order to determine if they have the potential to be utilized as liquid-biopsy based biomarkers for early diagnosis of HCC on the background of cirrhosis. β-actin expression was used as an internal control for normalization. All the samples were run in replicates for each gene to analyze their expression levels in cirrhosis as well as in HCC patients.

**Fig 3:**
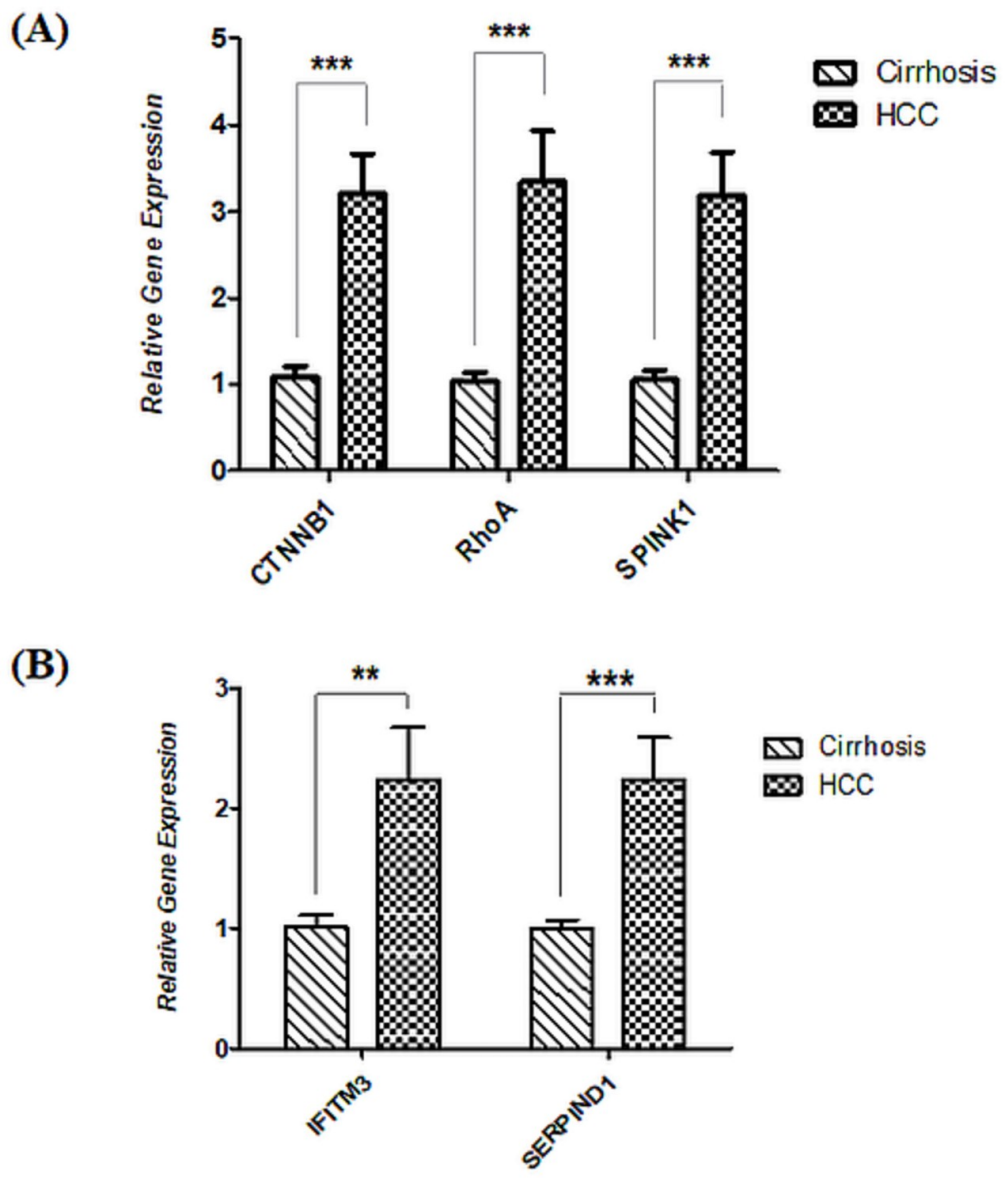
Relative expression analysis of selected gene signature in HCC patients as compared to cirrhotic patients. β-actin was used as internal control. (A) CTNNB1, RhoA and SPINK1 showed a highly significant increase of 3.32-,3.2- and 3.18-folds, respectively, in HCC patients as compared to cirrhosis patients while (B) IFITM3 and SERPIND1 were also significantly increased by 2.24-folds, each. Statistical analysis was done via two-tailed t-test using 95% CI. (***p value <0.001; **p value < 0.01).

The expression analysis of selected genes in terms of fold change in expression levels among cirrhosis and HCC patients is shown in Fig 3A and 3B. Statistical analysis of obtained results demonstrated that, RhoA, CTNNB1, and SPINK1 showed great potential to be used as biomarker for comparison of late-stage hepatic cirrhosis and early-stage HCC having cirrhotic background with a highly significant 3.34, 3.2 and 3.18-fold change while IFITM3 and SERPIND1 also gave significant results, both showing 2.24-fold change.

### ROC curve analysis of selected biomarkers

**Fig 4:**
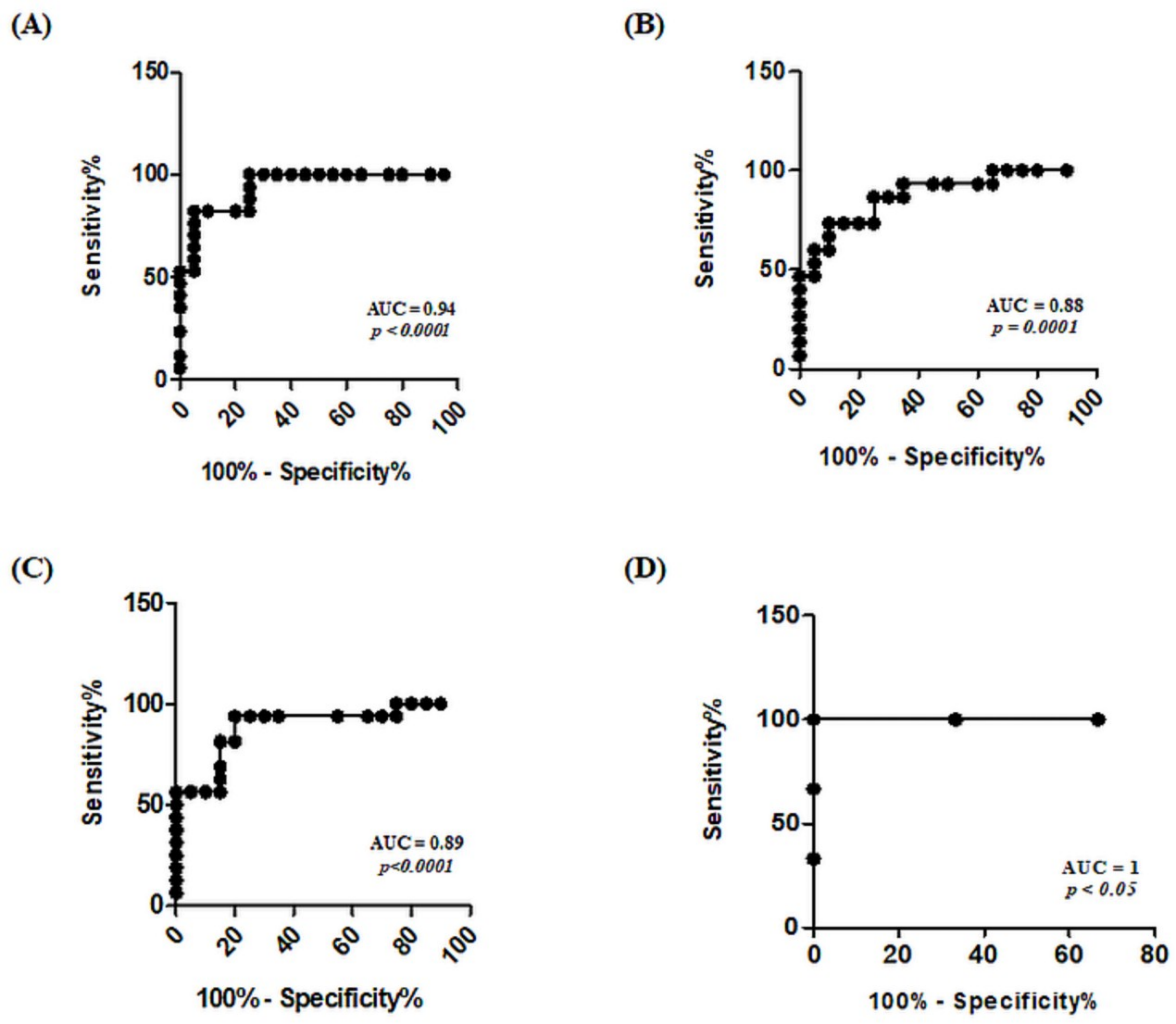
ROC curve analysis of selected genes (CTNNB1, SERPIND1, SPINK1) exhibiting better diagnostic ability. (A) ROC curve of CTNNB1 showed that it can detect HCC with 0.94 AUC having a p-value of <0.0001. (B) ROC curve analysis showed that SERPIND1 can detect HCC with 0.88 AUC having a p-value of 0.0001. (C) ROC curve of SPINK1 showing its ability to differentiate HCC from cirrhosis with 0.89 AUC and a p-value of <0.0001. (D) ROC curve analysis of RNA signature involving CTNNB1, SERPIND1, SPINK1 combined having AUC value of 1 with a p-value < 0.05.

The receiver operating characteristic (ROC) curve is regarded as an accurate summary of diagnostic capability of a biomarker. Sensitivity and specificities were plotted at y-axis and x-axis respectively. Cut-off value was set at 0.5 as standard. ROC curve analysis of HCC biomarkers used in this study indicated that CTNNB1, SERPIND1 and SPINK1 have better diagnostic values with AUC values as 0.94 (95% CI = 0.87–1.01, p<0.0001), 0.88 (95% CI = 0.76–0.99, p = 0.0001) and 0.89 (95% CI = 0.77–1, p<0.0001), respectively, as shown in Fig 4. A combination of these three biomarkers gives a highest AUC of 1 thus depicting their strong potential as diagnostic entities for diagnosing early-stage HCC from cirrhotic nodules.

## Discussion

HCC usually develops from underlying cirrhosis and the major issue in its late diagnosis is the inability to differentiate or predict the development of tumor aside from cirrhotic nodules. Therefore, there is lack of preventive strategies that could limit the disease at cirrhotic stage. As our knowledge on dual nature of platelets has also expanded in the recent years, they not only have protective role in our body but their bidirectional communication with tumor also affects the growth and metastasis of tumor [24]. Tumor cells have also been reported to impose changes on the RNA and protein content of platelets [25].

Emerging evidence of using TEPs in cancer diagnostics such as for colorectal cancer, non-small cell lung carcinoma, pancreatic cancer, hepatobiliary cancer, breast cancer and glioblastoma suggests its potential to be explored for other cancer types as well [15]. Another study by Calverley et al. demonstrated the potential of platelets to distinguish healthy donors from lung cancer patients by hierarchical clustering showing differential RNA expression in patients [26]. In another study done by our research group [16], we assessed the potential of TEP’s mRNA to detect HCC as compared to healthy controls which demonstrated significant results.

In current study, however, we have utilized the differential expression of TEPs’ mRNA signature for earlier diagnosis of HCC solely developing on the background of cirrhotic nodules and for the prediction of disease progression from cirrhosis to HCC. The selected mRNA included CTNNB1, SERPIND1, SPINK1, SERPIND1 and IFITM3 based on their predicted involvement in development of HCC on cirrhotic background. ROC curve analysis of these biomarkers showed promising diagnostic value but CTNNB1, SERPIND1 and SPINK1, with AUC as 0.94, 0.88 and 0.89, respectively, demonstrated that they have highest diagnostic ability to detect early stage HCC developing on the background of cirrhosis.

Our results showed a highly significant difference in expression of RhoA among cirrhosis and early-stage HCC patients. RhoA signaling is involved in cirrhosis driven carcinogenesis via LPA pathway as key regulator [27]. RhoA is reportedly known to be involved in stress fiber formation in liver cirrhosis and adhesion complexes formation [28]. Moreover, it contributes to the changes in the shape of platelets during their activation [29]. Thus their possible interaction is evident and predicted to be involved in HCC progression, hence the increase in expression. Our results are in agreement with the studies by Fukui et al. and XR Li et al. which reported the increased expression of RhoA as a predictor of recurrence and poor prognosis in early-stage HCC patients [30, 31]. The ROC curve analysis of RhoA had 0.75 AUC depicting its diagnostic value above cut-off i.e. 0.5. Our findings of increased RhoA expression are in accordance with previous findings. However, our study is the first to report this gene for molecular differentiation of cirrhosis and HCC patients using TEPs-based biopsy.

As Wnt/β-catenin signaling pathway is involved in the embryonic development and metabolic control in the liver, this oncogenic pathway is activated in most HCCs either due to CTNNB1 mutations or overexpression of nonnuclear type β-catenin such as reported by Wong et al. [32]. Thus, in order to analyze the involvement of this pathway in progression of cirrhosis to HCC, we assessed the transcriptomic expression of CTNNB1 in both study groups. Our results depicted a highly significant increase in CTNNB1 expression that can be attributed to the mutated Wnt pathway. This pathway causes nuclear translocation of β-catenin in HCC and increased expression which ultimately leads to regulation of the cell proliferation genes and increased cancer progression [33]. The CTNNB1 ROC curve was plotted which resulted in highest AUC of 0.94 with 95% CI = 0.87–1.00, further strengthening its diagnostic value.

SPINK1 is also known to mediate the growth and differentiation of tumor via suppression of apoptotic pathway in cancer cells and protecting them from immune surveillance [34]. The 3.2-folds increase of SPINK1 in HCC patients compared to cirrhosis in our study corelates with the findings of Lee et al. which suggests its role in increased metastatic potential and advancement of HCC. They also proposed it as marker for molecular staging and early tumor recurrence predictor [35]. Previously, the increase in SPINK1 gene expression in HCC as compared to HBV-induced cirrhotic patients has been reported [36]. Our study mostly included HCV-induced cirrhotic patients followed by HCV-induced cirrhosis patients but generating similar results with increased SPINK1 transcript correlating HCV-mediated cirrhosis and the risk of its progression to HCC. The ROC curve analysis of SPINK1 also showed higher AUC of 0.89 highlighting a very good sensitivity as well as specificity [37].

Recently, IFITM3 has been suggested to activate p38/MAPK pathway leading to the regulation of MMP9, which is involved in HCC invasion and metastasis [38]. Knockdown of IFITM3 expression has reportedly caused suppression of cancer growth indicating its involvement in tumor progression [39]. Therefore, the 2.24-fold increased expression of IFITM3 in HCC as compared to cirrhosis, demonstrated in our study, can also be attributed to its antiviral signaling and inflammation which are the hallmarks of tumor progression in cirrhosis and HCC cases having viral etiology. The ROC curve also demonstrated a 0.79 AUC which is above cut-off thus showing that it has potential as a good diagnostic entity. SERPIND1 is involved in coagulation, inflammation and cancer metastasis. It maintains homeostasis which suggests its potential interaction with platelets while contributing in coagulation and cancer metastasis. A previous study has reported its upregulation in liver cancer stem cells (CSCs) [40] which supports our results of its enhanced expression in HCC. Its high AUC value i.e. 0.88 with 95% CI = 0.76–0.99 in ROC curve analysis revealed its great potential to be used as a diagnostic biomarker. A combination of CTNNB1, SERPIND1 and SPINK1 would generate an AUC of 1 which would be the ideal scenario for early-stage HCC detection when developing from cirrhosis.

The crosstalk between platelets and tumor cells contribute to varying RNA transcripts in platelets of HCC patients as compared to platelets from hepatic cirrhosis patients. The biomarkers might be among the particles released by tumor cells or within tumor microenvironment and translocated to platelets hence increased expression is observed along with the progressive disease state. Moreover, in lieu of the differential expression of CD41 on platelets of cirrhosis and HCC patients, further confirmatory studies need to be carried out to confirm the hypothesis of its involvement in HCC. Positive and consistent results can prove CD41 to be a significant marker for diagnosing HCC at early stages in cirrhotic patients.

## Conclusion

The findings of our study suggest that expression levels of TEPs mRNA can be a useful liquid biopsy-based tool for earlier diagnosis of HCC developing on the background of underlying cirrhosis. Our suggested mRNA signature including CTNNB1, RhoA, SPINK1, IFITM3 and SERPIND1, alongside other platelets mRNA, can be utilized as biomarkers for comparison of hepatic cirrhosis and HCC. Moreover, differential expression of CD41 on platelets among cirrhosis and HCC patients may be associated with disease status hence further studies must be carried out to explore the probable association. The promising results of this study should be further validated by conducting the study on a greater number of samples in order to check the potential of this mRNA signature for liquid biopsy of HCC and explore if there is a link between CD41 expression on platelets and the disease.

Overall, our study provides a basis for the development of a liquid biopsy-based RNA signature for the detection of HCC as the altered RNA expression in HCC patients correlate with the findings of other TEPs-based studies. It paves the way for further exploration of RNA expression and receptors which could lead to understanding of various signaling pathways involved in progression of cirrhosis towards HCC. This can also aid in the development of therapeutic strategies to prevent emergence of HCC from cirrhotic nodules.

## Supporting information

S1 Table Set of primers (taken from published literature) used in the study. Primer sequences used in Real-time expression analysis for the corresponding genes of interest.
(DOCX)
