# Supplementary material for: Platelets’ RNA as biomarker trove for differentiation of early-stage hepatocellular carcinoma from underlying cirrhotic nodules
Source: PLoS One. 2021 Sep 1;16(9):e0256739. doi: 10.1371/journal.pone.0256739 (PMC8409664; doi:10.1371/journal.pone.0256739)
Supplement: S1 Table — Primer sequences used in Real-time expression analysis for the corresponding genes of interest. (DOCX) [file pone.0256739.s001.docx]

**S1 Table. Set of primers (taken from published literature) used in the study.** Primer sequences used in Real-time expression analysis for corresponding genes of interest.

| GENES | PRIMER SEQUENCE | PRODUCT SIZE |
| --- | --- | --- |
| β-actin F | ATCAAGATCATTGCTCCTCCTGA | 102bp |
| β-actin R | CTGCTTGCTGATCCACATCTG |  |
| IFITM3 F | ATGTCGTCTGGTCCCTGTTC | 184bp |
| IFITM3 R | GTCATGAGGATGCCCAGAAT |  |
| CTNNB1 F | AAAGCGGCTGTTAGTCACTGG | 132bp |
| CTNNB1 R | GACTTGGGAGGTATCCACATCC |  |
| RhoA F | GTGCCAAGGCGAAGTAAAAG | 200bp |
| RhoA R | AGTCGCCAACCAACACTTTC |  |
